# Isolation, purification, and characterization of polysaccharides from *Dendrobium findlayanum*: biological activities and potential therapeutic applications

**DOI:** 10.3389/fnut.2025.1740458

**Published:** 2025-12-12

**Authors:** Ji Chen, Linhong Li, Xu Mo, Lijuan Wu, Chaowen Zhang, Yu Sun, Yao Liu, Rui Li, Yuanfeng Zou, Yan Zheng, Fan Liu, Mengliang Tian

**Affiliations:** 1 College of Agronomy, Sichuan Agricultural University, Chengdu, China; 2 Leshan Academy of Agricultural Sciences, Leshan, China; 3 Natural Medicine Research Center, College of Veterinary Medicine, Sichuan Agricultural University, Chengdu, China; 4 Department of Medical Plastic and Cosmetic, The Third People’s Hospital of Chengdu (The Affiliated Hospital of Southwest Jiaotong University), College of Medicine, Southwest Jiaotong University, Chengdu, China

**Keywords:** *Dendrobium findlayanum* polysaccharides, inflammation, intestinal porcine enterocyte cells, oxidative stress, structural characterization

## Abstract

The crude polysaccharides of *Dendrobium findlayanum* were extracted by ethanol precipitation and separated using DEAE Sepharose Fast Flow and Sepharose 6 Fast Flow chromatography to obtain DFP-NP and its purified fraction DFP-NP1. Structural characterization revealed that DFP-NP (12.3 kDa) primarily consisted of mannose, rhamnose, glucose, and galactose in a molar ratio of 11.61:0.68:84.78:2.93, with FT-IR spectra confirming typical polysaccharide functional groups. *In vitro* assays demonstrated that DFP-NP1 polysaccharides at concentrations of 5.0–20 μg/mL enhanced CAT and SOD activities and upregulated related gene expression in IPEC-J2 cells, while reducing TNF-α, IL-1β, and IL-6 secretion. They decreased expression levels of genes associated with the antioxidant signaling and cellular inflammatory pathways in IPEC-J2 cells. Therefore, *D. findlayanum* polysaccharides possess significant potential for application as a nutraceutical supplement to alleviate intestinal inflammation linked to oxidative stress, leveraging a dietary approach for health maintenance.

## 1 Introduction

*Dendrobium* (*D.*), an epiphytic herb belonging to the Orchidaceae family, 76 species native to China with three-quarters demonstrating medicinal properties (1–4). *Dendrobium* exhibits multiple biological activities, including anti-fatigue, antioxidant, anti-inflammatory, antibacterial, anti-tumor, anti-aging, anticoagulant, antihypertensive, blood sugar regulation, immune enhancement, and cognitive function modulation (5–7). As a traditional and valuable medicinal herb, *Dendrobium* contains multiple active ingredients, including alkaloids, sesquiterpenes, flavonoids, coumarins, benzyl benzoate, phenols, polysaccharides, and other compounds (8). *Dendrobium* polysaccharides, a primary active component of *Dendrobium*, play a key role in its pharmacological action, especially in anti-inflammatory and immunomodulatory aspects with potential applications.

Polysaccharides are natural macromolecules formed by the condensation of several monosaccharides, which are widely found in nature (9). *Dendrobium* polysaccharides exhibit diverse biological activities, including antioxidant, anti-inflammatory, anti-tumor, anti-bacterial, and immune regulatory functions (10–13). The activities of *Dendrobium* polysaccharides are significantly influenced by species, molecular structure, extraction methods, and others (14–16). Variations in growing conditions and extraction techniques can alter polysaccharide composition and structure, subsequently influencing their biological activities (17–19). Polysaccharides obtained from *D. candidum* using ultrasonic extraction showed higher activity than water extraction (20); moreover, polysaccharides with different molecular weights (MW) showed significant differences in anti-tumor and anti-inflammatory activities (21). Therefore, it is essential to investigate further the biological activities and mechanisms of *Dendrobium* polysaccharides for their medicinal development.

Currently, inflammatory bowel disease (IBD), encompassing a range of chronic and recurring inflammatory conditions of the digestive tract, has become a major global health concern, profoundly affecting the quality of life of patients (22). IBD, which includes Crohn’s disease and ulcerative colitis as its main types, has a complex etiology, which may involve a variety of factors such as dysregulation of the immune system, imbalance of intestinal microorganisms and the environment (23). Oxidative stress is intricately linked to IBD; it is both one of the potential triggers and an essential driving factor in the pathogenesis of IBD. Oxidative stress leads to an overproduction of reactive oxygen species (ROS), and the excess of ROS induces the secretion of large quantities of pro-inflammatory factors by pro-inflammatory cells (24, 25), and at the time of the onset of IBD, the patient’s immune tolerance to intestinal microorganisms decreases, resulting in an excessive inflammatory response characterized by elevated levels of pro-inflammatory factors such as IL-23, TNF-α, and IL-6 (26), this surge in pro-inflammatory cytokines further damages the intestinal tract and intensifies the inflammation. Corticosteroids, immunosuppressants, and biologics are commonly employed in IBD treatment (27–29), but their long-term use can bring significant side effects such as skin disorders, osteoporosis, and loss of appetite (27, 30), there has been gradual attention to natural plant components as potential therapeutic options. It has been shown that the anti-inflammatory, immunomodulatory and antioxidant activities of *Dendrobium* polysaccharides can produce positive effects on alleviating IBD symptoms, which provides new research ideas for their application in IBD treatment (31, 32).

Research predominantly targets widely cultivated *Dendrobium* species like *D. officinale*, *D. huoshanense*, *D. candidum*, and *D. nobile*, despite the genus’s diversity (33–35). Research on other *Dendrobium* species is relatively limited. *D. findlayanum* is native to Yunnan’s mid-altitude forests (800–900 m) and Southeast Asia, whose enlarged internodes present a unique morphological advantage for polysaccharide accumulation. However, no systematic analysis exists regarding its polysaccharide profiles or their therapeutic relevance to inflammatory conditions, establishing this investigation as the first comprehensive exploration of *D. findlayanum’s* most abundant yet functionally unknown components (36). Five alkaloids extracted from the stem of *D. findlayanum* show inhibitory effects on the growth of tumor cells (37). However, its polysaccharides’ composition, structure and biological functions have yet to be thoroughly studied.

This study pioneers the systematic characterization of *D. findlayanum* polysaccharides (DFP) through gradient ethanol precipitation and DEAE cellulose chromatography, elucidating their monosaccharide composition and glycosidic linkage patterns via GC–MS and NMR. Using a porcine intestinal epithelial cell inflammation model, we evaluated these polysaccharides’ potential anti-inflammatory and protective effects. The results of the study show that *D. findlayanum* polysaccharides provide a good basis for practical applications and support the future development of *D. findlayanum* polysaccharide-based food supplement formulations or as adjunctive therapies for the treatment of intestinal disorders and other inflammation-related diseases.

## 2 Materials and methods

### 2.1 Materials and reagents

*D. findlayanum* was collected from Mangshi, Dehong Prefecture, Yunnan Province, and identified as *D. findlayanum*. The samples underwent washing, and thermal treatment at 105 °C for 30 min, drying at 50 °C until a constant weight was achieved, crushing, sieving through a 60-mesh sieve, and were then stored in a dry environment for further use.

DEAE Sepharose Fast Flow and Sepharose 6 Fast Flow were obtained from Beijing RuiDaHengHui Science & Technology Development Co., Ltd., China. Enzyme-linked immunoassay (ELISA) kits were obtained from Shanghai Kexing Trading Co., Ltd., China. CCK-8 kits and 3,500 Da dialysis bags were from Solarbio, China. All analytical-grade reagents were supplied from Chengdu Chron Chemical Co., Ltd., China.

### 2.2 Isolation and purification of polysaccharides

Sixty grams of *D. findlayanum* powder was weighed and added to 95% alcohol solution and extracted for 2 h to remove the pigmentation. This extraction was repeated four times. The residue was dried in an oven at 50 °C after filtering the solution. The dried powder was extracted with distilled water (40 volumes) at 80 °C for 2 h. The filtrates were concentrated to about 100 mL using a rotary evaporator at 50 °C and then dialyzed with a 3,500 Da MW cutoff dialysis bag. Following dialysis, anhydrous ethanol was added in a 4:1 ratio, and the resulting precipitate was collected and lyophilized to yield the crude polysaccharides, referred to as DFP. After dialysis, anhydrous ethanol was added in a ratio of 4:1, and the mixture is allowed to precipitate at 4 °C for 24 h. The precipitate is collected and freeze-dried (Foring Technology Development Ltd., China) to obtain crude polysaccharide, referred to as DFP. DFP was dissolved in deionized water and applied to a DEAE Sepharose Fast Flow anion exchange column. The column was eluted with ultrapure water at a 2 mL/min flow rate to collect the neutral polysaccharides (called DFP-NP), collecting 10 mL fractions. Acidic polysaccharides were eluted with a gradient of sodium chloride solution at a concentration of 0–1.5 M. However, since the acidic sugars were less abundant, this study concentrated on neutral polysaccharides. The samples were further purified using a Sepharose 6 fast flow column, eluting with ultrapure water at a flow rate of 0.5 mL/min and collecting each 2 mL fraction. The purified neutral polysaccharide was named DFP-NP1.

### 2.3 Determination of MW

Gel permeation chromatography (GPC) coupled with multi-angle scattering was used to determine the MW of the purified polysaccharides. The liquid phase system is U3000 (Thermo, United States) and the laser light scattering detector is DAWN HELEOS II (Wyatt technology, CA, United States). Five milligram polysaccharide sample was precisely weighed and dissolved in 1 mL of 0.1 mol/L sodium nitrate solution, yielding a 5 mg/mL polysaccharide solution. Poly (ethylene oxide) with a MW of 18.67 kDa was used as a standard and was utilized at a concentration of 5 mg/mL. The system operated with an injection volume of 100 μL, eluted using a 0.9% (w/v) NaCl solution at 0.5 mL/min, and maintained at 30 °C.

### 2.4 Chemical composition and monosaccharide composition analysis

The phenol-sulfuric acid method was employed to quantify the obtained reducing polysaccharides, the Folin-Phenol method to determine total phenols, and the Coomassie Brilliant Blue G-250 method to quantify total protein. Monosaccharide composition was analyzed using PMP-HPLC, the mixture was heated to 70 °C for 1 h for derivatization, then cooled to room temperature and neutralized with 400 μL of 0.3 mol/L HCl. The solution was combined with 0.5 mL of 3 M HCl and 0.5 mL of CHCl3, followed by centrifugation at 3,000 rpm for 5 min. This extraction process is repeated three times until the lower phase becomes colorless, and the solution is filtered through a 0.22 μm microporous membrane. Monosaccharide standards are prepared, and the aqueous phase is retained as a control. The hydrolysate of polysaccharides and standards of different concentrations are analyzed using high-performance liquid chromatography (Agilent, United States), employing a ZORBAX SB-C18 column (250 mm × 4.6 mm, 5 μm) for analysis, with a gradient elution of acetonitrile (A) and 0.05 mol/L phosphate buffer (pH 6.7) (B).

### 2.5 FT-IR and NMR analyses

A 1 mg sample of dried DFP-NP1 was combined with potassium bromide, ground in an agate mortar, and pressed into transparent slices. An FT-IR spectrometer scanned these samples from 4,000 cm^−1^ to 400 cm^−1^. DFP-NP1 was subjected to three cycles of dissolution in deuterium oxide (D_2_O, Sigma-Aldrich) followed by lyophilization to ensure complete deuterium exchange. The NMR spectrometer (Bruker Avance III HD 800 MHz, Rheinstetten, Germany) was calibrated using 2,2,3,3-tetradeuterated-3-(trimethylsilyl) propionic acid sodium salt (TSP-d4) as internal reference. ^1^H, ^13^C, HSQC, and HMBC spectra were acquired at 60 °C (38, 39). Data processing was performed with MestReNova software (v14.2, Mestrelab Research).

### 2.6 Determination of the anti-inflammatory activity of *Dendrobium findlayanum* polysaccharides

#### 2.6.1 Cell culture

Frozen cells from the −80 °C refrigerator were quickly thawed. The cells were transferred to a Petri dish containing 9 mL of complete medium, dispersed, and incubated at 37 °C, 5% CO_2,_ and 90% relative humidity. When the cells were full, the cells were washed twice with 4 mL of PBS buffer, digested with 2 mL of PBS buffer and 1 mL of Trypsin–EDTA (without phenol red) for 3 min, and then the digestion was stopped by adding 4 mL of DMEM medium. The adherent cells were completely dislodged by pipetting. The cytotoxic effects of DFP-NP1 from the size exclusion column and lipopolysaccharide (LPS; Sigma-Aldrich) on IPEC-J2 cells were evaluated through CCK-8 assay (Dojindo, Japan) following 24-h exposure at concentrations ranging from 5 to 100 μg/mL. Cells were incubated with various concentrations of DFP-NP1 (5, 10, 20, 40, 80, and 100 μg/mL) in a 96-well cell for 24 h. Then added CCK-8 reagent to detect viability.

#### 2.6.2 The pro-inflammatory response in IPEC-J2 cells triggered by lipopolysaccharide

The cell was cultured for 24 h, then rinsed with 1 mL of PBS, and then DFP-NP1 solution was added and cultured for another 24 h. The preventive experimental groups included the blank control group (medium only), model group (20 μg/mL LPS), and DFP-NP1 + LPS group (5, 10, 20 μg/mL DFP-NP1 and LPS).

The cell suspension was distributed into 6-well plates, and 1 mL was added to each well. After 24 h of incubation, the cells were rinsed twice with 1 mL of PBS, and a complete medium containing 20 μg/mL LPS was added and incubated for 24 h. The treatment experimental groups included: blank control group: medium only; model group: only 20 μg/mL LPS solution was added as the final concentration; DFP-NP1 + LPS group: 20 μg/mL LPS solution was added and incubated for 24 h. After 24 h of incubation, different concentrations of DFP-NP1 solution (5, 10, 20 μg/mL) were added and incubated for 24 h.

Elisa assay: Determine the content of TNF-α, IL-1β, IL-6, SOD, CAT by referring to the kit instructions. RNA extraction: RNA was extracted by TRIzol method, which was used to detect the expression level of mRNA and the expression level of related pathway genes. The primers are shown in the Supplementary Table S1.

## 3 Results and discussion

### 3.1 Isolation, purification, and MW determination of *Dendrobium findlayanum* polysaccharides

**Figure 1:**
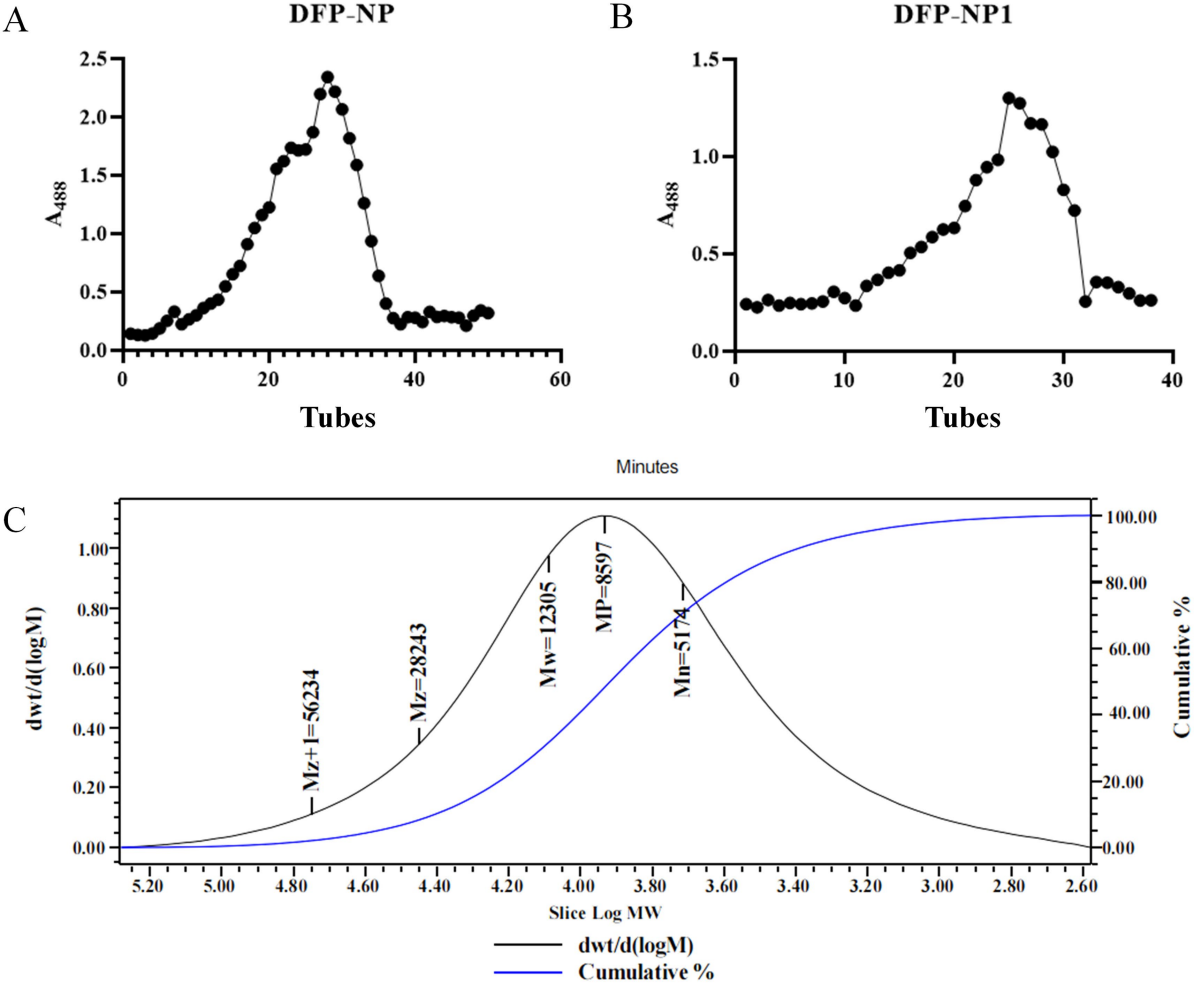
Isolation and purification of polysaccharides from *Dendrobium findlayanum*. Wash elution curve of DFP-NP on DEAE Sepharose Fast Flow anion exchange column **(A)**; Purification curve of DFP-NP1 on Sepharose 6 Fast Flow column **(B)**; GPC chromatogram of DFP-NP polysaccharide **(C)**.

Anion exchange chromatography facilitates the separation of neutral and acidic polysaccharides, enabling preliminary isolation and purification of polysaccharides (40). A neutral polysaccharide fraction (DFP-NP) was isolated from *D. findlayanum*, resulting in a single peak in the elution profile (Figure 1A). The collected neutral sugar fraction was concentrated and lyophilised, then purified using a Sepharose 6 Fast Flow column. The resulting purification curve revealed a single, homogeneous component designated as DFP-NP1 (Figure 1B).

Gel permeation chromatography (GPC) is an effective technique for analysing high MW soluble organic compounds, including proteins and polysaccharides (41–43). However, the complex structure of polysaccharides and the presence of isomers often lead to insufficient identification when using chromatography alone (44). To address this limitation, GPC is frequently coupled with multi-angle laser light scattering (MALLS) to comprehensively characterize high MW compounds (45–47).

In this study, we employed GPC-MALLS to determine the relative molecular mass of DFP-NP. The results revealed that the weight-average MW of DFP-NP was 12.3 kDa (Figure 1C). The GPC chromatogram exhibited a single peak, indicating that the obtained *D. findlayanum* polysaccharide is homogeneous. It is worth noting that the molecular weight of DFP-NP was significantly lower than that of common *Dendrobium* polysaccharides, such as those isolated from *D. Huoshanense* with molecular weights ranging from 22 to 8,090 KDa, *D. officinale* polysaccharides with molecular weights often in the range of 130–952 KDa, and *D. nobile* polysaccharides with molecular weights often in the range of 11.4–710 KDa (34), and lower molecular weights may be correlated with their activities. The low molecular weight of DFP-NP1 (12.3 kDa) enhances its immunomodulatory potential by increasing solubility and cellular uptake. However, no comparative activity data were detected in the current experiments, and subsequent experiments are needed to verify the effect of molecular weight on activity.

### 3.2 Chemical composition and monosaccharide analysis of *Dendrobium findlayanum* polysaccharides

Polysaccharide composition is essential in defining their physical, chemical, and biological characteristics (48, 49). We analyzed the content of total sugars, proteins, and polyphenols in crude polysaccharides (DFP), neutral polysaccharides (DFP-NP), and purified neutral polysaccharides (DFP-NP1) extracted from *D. findlayanum*. The polysaccharide content in DFP, DFP-NP, and DFP-NP1 was 39.7 ± 2.2%, 65.4 ± 1.7%, and 87.6 ± 4.7%, respectively. Protein content was 16.2 ± 3.2%, 5.7 ± 1.9%, and 2.3 ± 0.8%. Polyphenols also affect anti-inflammatory activity (50), the polyphenol content of the three polysaccharides was examined and found to be relatively low at 1.4 ± 0.4%, 0.8 ± 0.2% and 0.5 ± 0.2%, respectively, which excludes the effect of more polyphenol content on the results of the experiment. Consistent with studies on other *Dendrobium* species such as *D. officinale* (51) and *D. huoshanense* (52) the monosaccharide composition of *D. findlayanum* polysaccharides primarily consisted of glucose and mannose, accounting for 96.39% of the total content. Small amounts of galactose and rhamnose were also detected. The molar ratio of glucose:mannose:galactose:rhamnose was 84.78:11.61:2.93:0.68, with a glucose to mannose ratio of 7.3:1.

Different monosaccharides contribute to various functional properties of polysaccharides; glucose and mannose influence prebiotic and immunomodulatory activities, while rhamnose affects antioxidant activity (53). The high proportion of glucose and mannose observed in this study may contribute to the bioactivity of *D. officinale* polysaccharides, a finding consistent with previous studies on *D. huoshanense* (54), *D. chrysotoxum* (55), and *D. nobile* (56).

### 3.3 Functional group analysis of *Dendrobium findlayanum* polysaccharides using FT-IR

**Figure 2:**
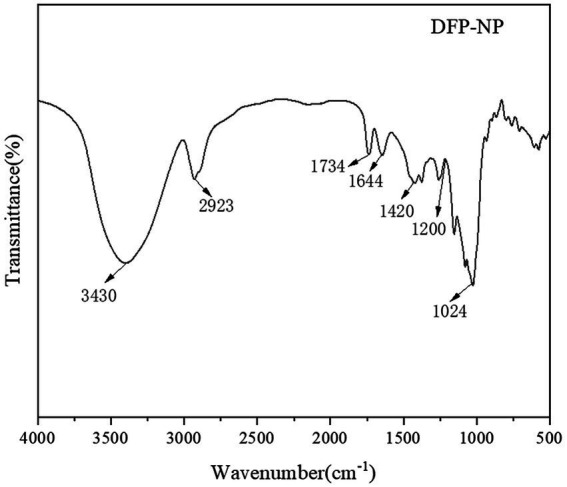
FT-IR spectrum of DFP-NP.

The functional groups potentially present in *D. findlayanum* polysaccharides were analyzed based on their infrared spectroscopy profile. As illustrated in Figure 2, DFP-NP exhibited an absorption peak at 3430 cm^−1^, characteristic of O-H and N-H stretching vibrations. The absorption peak at 2923 cm^−1^ was attributed to C-H stretching vibrations. These two functional group regions are typical absorption peaks for polysaccharides (57).

C=O vibration at 1734 cm^−1^ and the absorption peaks of acetamido and C=O at 1644 cm^−1^ (58). The region between 1,420 and 1,200 cm^−1^ was associated with C-H bending vibrations (59, 60). The peak at 1,024 cm^−1^ was attributed to −CHO stretching vibrations, which are characteristic of pyranose ring structures (61, 62). This observation suggests the presence of pyranose glycosidic bonds in DFP-NP.

Pyranosidic bonds play a crucial role in determining the structure and stability of polysaccharides and in influencing their biological activities (63). The specific linkage patterns of pyranosidic bonds, such as 1 → 4 or 1 → 6 connections, have been shown to significantly impact the molecular structure and stability of polysaccharides (64). Moreover, several studies have reported a potential correlation between pyranosidic bonds and the antioxidant properties of polysaccharides (65). Given the presence of pyramidic bonds in *D. findlayanum* polysaccharides, it can be postulated that these compounds may exhibit substantial biological activities. However, further investigation is necessary to elucidate the precise relationship between the pyranosidic bond configuration and the specific biological functions of these polysaccharides.

### 3.4 Structural characterization of *Dendrobium findlayanum* polysaccharides using NMR spectroscopy

**Figure 3:**
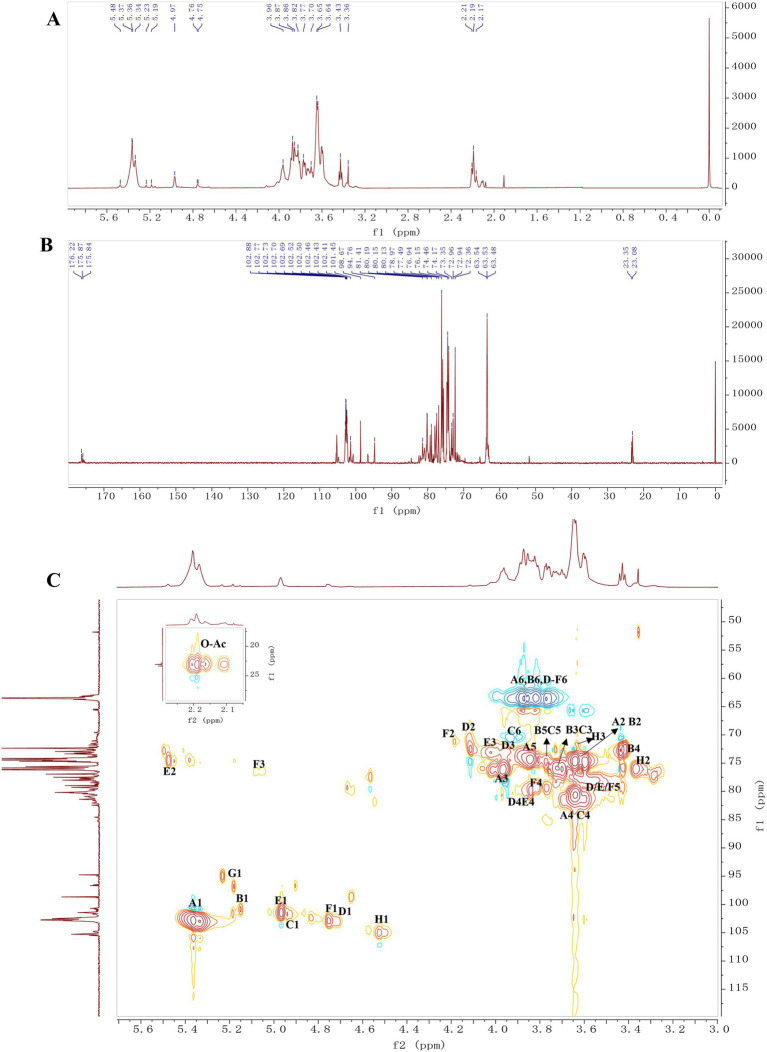
Structural characterisation of *Dendrobium findlayanum* polysaccharides (DFP-NP1): **(A)** ^1^H-NMR, **(B)** ^13^C-NMR and **(C)** HSQC spectra of DFP-NP.

To further elucidate the chemical structure of DFP-NP1, nuclear magnetic resonance (NMR) analysis was conducted. The ^1^H NMR spectrum revealed three significant peaks in the *δ* 5.19–5.48 ppm region (Figure 3). At 5.18 ppm, 5.23 ppm, and 5.37 ppm, these anomeric proton signals indicated α-configurations for three residues. Comprehensive analysis of ^1^H, ^13^C, and HSQC spectra allowed the assignment of residue G (*δ* 5.18/96.8 ppm, 5.23/95.0 ppm) to →4)-α-Manp and residue A (*δ* 5.37/102.9 ppm) to →4)-α-Glcp-(1→. According to previously reports, the central peak at *δ* 4.94 also represented an α-configuration, corresponding to →4,6)-α-Glcp-(1 → (labeled as C). The signal observed at *δ* 5.15/100.9 ppm (residue B) was tentatively assigned to Glcp-(1→ (42, 66).

The ^1^H spectrum also showed signals in the *δ* 4.53–4.97 ppm range, indicating the presence of β-configurations in DFP-NP. Corresponding ^13^C signals at *δ* 101.5–105.1 ppm and HSQC analysis led to the assignment of residue D as →4)-β-Manp-(1 → and residue H as →4)-β-Glcp-(1→ (67–69). As shown in Table 1, residue E exhibited similar chemical shifts for H1/C1, H3/C3, and H5/C5 to residue D, with identical H4/C4 and H6/C6 shifts, suggesting a → 4)-β-Manp-(1 → structure. The H2/C2 chemical shift difference compared to residue D indicated a possible 2-*O*-Ac substitution. HSQC and HMBC spectra revealed *O*-Ac (O-CO-CH3) signals at *δ* 2.20/23.1 (176.0), 2.19/23.1 (176.2), 2.16/23.1 (175.8), and 2.11/23.1 (175.5), leading to the assignment of residue E as →4)-2-*O*-Ac-β-Manp-(1→ (12, 70). Similarly, residue F was identified as →4)-3-*O*-Ac-β-Manp-(1→, with *O*-Ac at C3. Detailed information is summarized in Table 1.

**Table 1:** Chemical shift assignment of DFP-NP (*δ*, ppm).

Residues		H1/C1	H2/C2	H3/C3	H4/C4	H5/C5	H6/C6
A	→4)-α-Glc*p*-(1→	5.37/102.9	3.64/74.6	3.97/76.1	3.64/80.6	3.85/74.3	3.93, 3.77/63.6
B	Glc*p*-(1→	5.15/100.9	3.59/74.8	3.70/76.1	3.44/72.8	3.81/74.4	3.93, 3.77/63.6
C	→4,6)-α-Glc*p*-(1→	4.94/101.7	3.64/74.6	3.69/76.1	3.60/81.4	3.77/74.8	3.90, 3.94/70.5
D	→4)-β-Man*p*-(1→	4.71/103.1	4.11/72.5	3.94/72.9	3.84/80.0	3.59/77.9	3.92, 3.77/63.5
E	→4)-2-*O*-Ac-β-Man*p*-(1→ *	4.97/101.5	5.48/74.7	4.02/73.3	3.84/80.0	3.61/77.9	3.92, 3.77/63.5
F	→4)-3-*O*-Ac-β-Man*p*-(1→ *	4.76/102.9	4.18/71.2	5.09/76.6 5.05/76.4	3.81/79.4	3.59/77.9	3.92, 3.77/63.5
G	→4)-α-Man*p*	5.23/95.0 5.18/96.8	3.58/n.d.	n.d.	n.d.	n.d.	n.d.
H	→4)-β-Glc*p*-(1→	4.53/105.1	3.37/76.12	3.63/71.6	3.81/79.3	n.d.	3.93, 3.77/63.6

*O-Ac (O-CO-CH3) was observed at δ 2.20/23.1 (176.0), 2.19/23.1 (176.2), 2.16/23.1 (175.8), 2.11/23.1 (175.5) in HSQC and HMBC spectra; n.d.: Not assigned.

### 3.5 Anti-inflammatory activity of *Dendrobium findlayanum* polysaccharides

**Figure 4:**
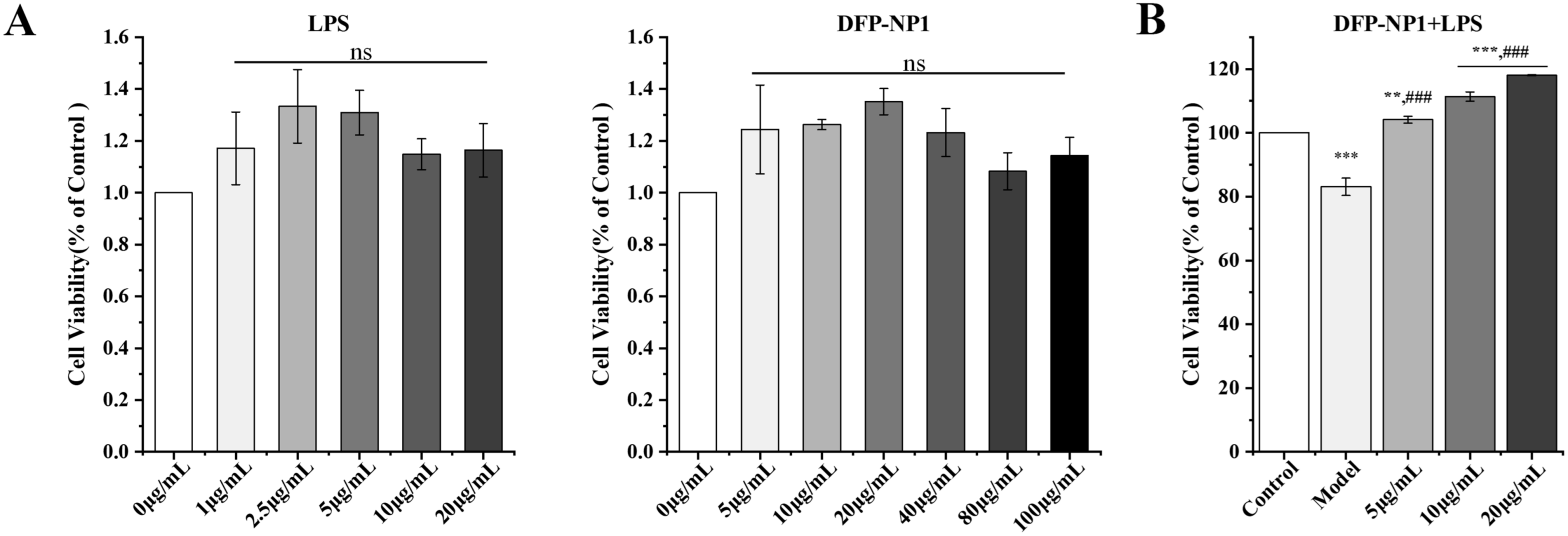
Cytotoxicity test. The effects of LPS and DFP-NP1 on cell viability **(A)**; Effect of LPS on cell viability after treatment with different concentrations of DFP-NP1 **(B)**. (*n* = 5) (* vs control; # vs model; ns represents no significant difference).

The CCK-8 assay assessed the survival of IPEC-J2 cells after exposure to different concentrations of LPS and DFP-NP1 for 24 h. LPS at concentrations of 0, 1, 2.5, 5, 10, and 20 μg/mL had no cytotoxic effect on IPEC-J2 cells, which is consistent with the sub-toxicity concentrations of LPS-induced inflammation in IPEC-J2 cells reported in previous studies (71). Consequently, an LPS concentration of 20 μg/mL was chosen to induce cellular inflammation in subsequent experiments. DFP-NP1 treatments at concentrations of 0, 5, 10, 20, 40, 80, and 100 μg/mL did not adversely affect IPEC-J2 cell viability. Notably, within the 0–20 μg/mL range, DFP-NP1 exhibited a dose-dependent increase in IPEC-J2 cell viability, with maximal viability observed at 20 μg/mL (Figure 4A). Based on these findings, polysaccharide supplementation was categorized into three groups: 20 μg/mL DFP-NP1 group, 10 μg/mL DFP-NP1 group, and 5 μg/mL DFP-NP1 group.

#### 3.5.1 Preventive effects of *Dendrobium findlayanum* polysaccharides on LPS-induced inflammation in IPEC-J2 cells

LPS induces pro-inflammatory factor secretion and oxidative stress, leading to increased ROS levels. Excessive ROS can compromise cellular structure and function (72, 73). The polysaccharides used in this experiment have been purified by ethanol precipitation, ion exchange chromatography and size exclusion chromatography, which have sufficiently reduced the LPS content to exclude the influence of LPS on the experimental results. To explore the preventive effects of *D. findlayanum* polysaccharides on LPS-induced inflammation, IPEC-J2 cells were pre-treated with different concentrations of DFP-NP1 prior to LPS exposure. The efficacy of different DFP-NP1 concentrations in preventing IPEC-J2 cell inflammation was evaluated by analysing oxidative stress levels and the expression of relevant inflammatory marker genes. The results showed that cell viability was significantly reduced in the LPS-induced model compared to the control group (*p* < 0.001), suggesting that inflammation impairs the structure and function of cells. Cell viability was increased in the DFP-NP1 prevention group compared to the model. Notably, 5 μg/mL DFP-NP1 significantly increased cell viability (*p* < 0.01), whereas 10 μg/mL DFP-NP1 resulted in a more pronounced increase (*p* < 0.001) (Figure 4B). A dose-dependent relationship was observed, with cell viability progressively increasing with increasing DFP-NP1 concentration, and the dose-dependent protective effect of DFP-NP1 may be directly related to its polysaccharide properties, with the low molecular weight property (12.3 kDa) potentially enhancing its transmembrane permeability to more efficiently neutralize ROS or block inflammatory signaling (74). These findings suggest that *D. findlayanum* polysaccharides attenuate LPS-induced cellular damage and enhance IPEC-J2 cell viability.

**Figure 5:**
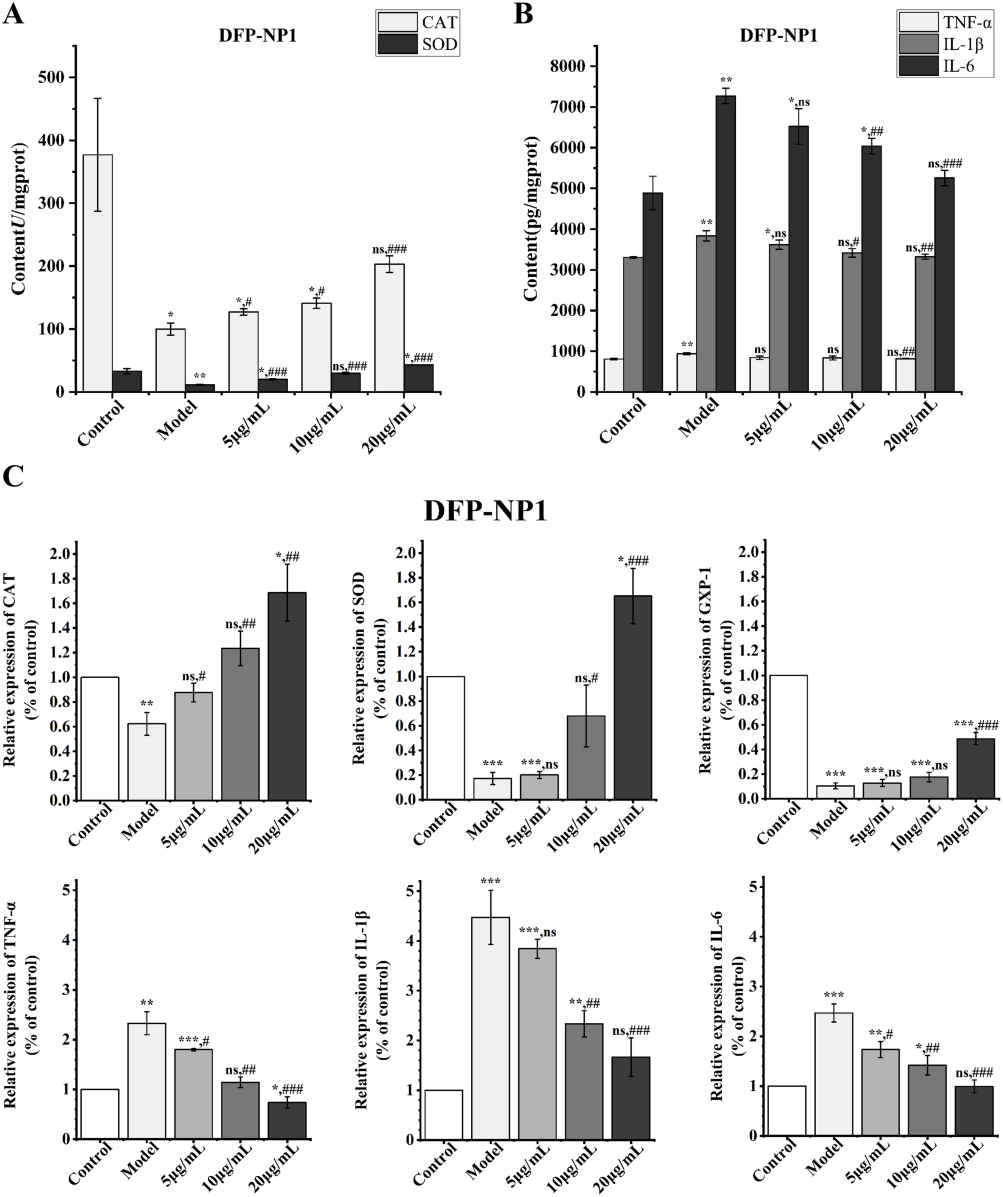
Effects of DFP-NP1 on the contents of CAT and SOD in IPEC-J2 cells **(A)**; Effects of DFP-NP1 on the contents of TNF-α, IL-1β, IL-6 in IPEC-J2 cells **(B)**; Effect of DFP-NP1 on the expression of *CAT*, *SOD*, *GXP-1*, *TNF-α*, *IL-1β*, *IL-6* **(C)**. (*n* = 5) (* vs control; # vs model; ns represents no significant difference).

SOD (*p* < 0.01) and CAT (*p* < 0.05) activities were significantly reduced in the LPS-induced IPEC-J2 cell model (Figure 5A), indicating that the inflammatory response triggers oxidative stress and impairs cellular antioxidant capacity. The model exhibited significantly elevated levels of IL-1β, IL-6, and TNF-α (*p* < 0.01), indicating a pronounced inflammatory response (Figure 5B).

In the 5, 10 μg/mL DFP-NP1 prevention groups, TNF-α levels were not clearly different from the models; however, at a concentration of 20 mg/mL, TNF-α levels were significantly decreased (*p* < 0.01). About IL-1β, it was significantly reduced in the 10 mg/mL DFP-NP1 (*p* < 0.05) and even more so in the 20 mg/mL (*p* < 0.01). In the 10 mg/mL DFP-NP1, IL-1β levels were significantly decreased and even more significantly in the 20 mg/mL (*p* < 0.01). For IL-6, there was no significant difference in the 5 μg/mL DFP-NP1, but the 5, 10 μg/mL DFP-NP1 clearly reduced IL-6 levels (*p* < 0.01), and IL-6 reached the lowest level at the highest concentration (*p* < 0.001).

High-temperature water can accelerate the diffusion rate of polysaccharides, thus improving the extraction efficiency (75). Polysaccharides are dispersed polymers that form intermolecular hydrogen bonds in aqueous solutions, and the addition of ethanol to polysaccharides solutions can lead to dehydration of polysaccharides (76), producing a white precipitate. Alcohol precipitation of polysaccharides with different concentrations of ethanol results in differences in the resulting polysaccharides’ size, structure, and activity (77–79). Precipitation of polysaccharides from *Cordyceps militaris* using different ethanol concentrations showed that lower ethanol concentrations resulted in higher MW, while higher ethanol concentrations led to a reduction in the MW of the polysaccharides (80). In this study, polysaccharides were alcohol precipitated using a polysaccharide solution: ethanol feed ratio of 1:4, and polysaccharides with a heavy average MW of 12.3 kDa were obtained, which was lower compared to *D. officinale* (81), *D. nobile* (82), *D. denneanum* (83) which were alcohol precipitated using the same ethanol concentration, and smaller MW may be the reason for the higher activity of DFP-NP (84, 85). From the results of 3.3, DFP-NP showed acetylation modification, which is more common in *D.* polysaccharides, and acetylation can change the structure of polysaccharides by exposing more groups, which may increase the antioxidant and immunomodulatory activities of polysaccharides (86).

In conclusion, polysaccharides from *D. findlayanum* restored the normal secretion levels of TNF-α, IL-1β and IL-6 in LPS-damaged IPEC-J2 cells, and various aspects influenced this favorable therapeutic effect.

The mRNA expression of *CAT*, *SOD*, and *GXP1* was detected to elucidate the molecular regulation of the antioxidant system by *D. findlayanum* polysaccharides during the inflammatory response in IPEC-J2 cells (Figure 5C). The models (LPS-stimulated) significantly reduced gene expression of *CAT*, *SOD*, and *GXP1* (*p* < 0.01), suggesting that oxidative stress leads to decreased expression of antioxidant enzymes and diminished antioxidant capacity. This was improved by DFP-NP1 treatment, where *CAT* and *SOD* reached their highest expression at 20 mg/mL DFP-NP1 (*p* < 0.01, *p* < 0.001) and *GXP1* was significantly increased only at 20 mg/mL DFP-NP1 (*p* < 0.001). The findings indicate that *D. findlayanum* polysaccharides enhance antioxidant enzyme gene expression, thereby exerting antioxidant effects.

The expression of *TNF-*α, *IL-1β*, and *IL-6* was also examined and the trends were consistent with the physiological content analysis. *TNF-*α was significantly upregulated in the model (*p* < 0.01) and *IL-1β* and *IL-6* were highly upregulated (*p* < 0.001). In contrast, DFP-NP1 treatment notably reduced cytokine expression, with 20 mg/mL DFP-NP1 leading to more pronounced decreases.

LPS can increase the permeability of intestinal epithelial cells and disrupt the epithelial barrier, which is prone to induce colitis due to bacterial infection (87). LPS disrupts the cellular barrier, causing intestinal dysfunction by producing pro-inflammatory factors. *Bletilla striata* polysaccharides, when applied at various concentrations to LPS-induced IEC-18 cell inflammation models, were found to inhibit the production of pro-inflammatory cytokines IL-6 and TNF-α (88). A study on *Dendrobium* found that *D. officinale* polysaccharides inhibited the proliferative activity of IL-22 induced hyperproliferative keratinocytes and treated LPS-induced inflammation (89). Also, in a model of inflammation resulting from LPS damage to IPEC-J2 cells, pretreatment with 100 mg/L *Astragali Radix* polysaccharides for 4 h significantly improved cell viability and boosted the activities of SOD and CAT enzymes, along with increasing the levels of IL-6, IL-8, and TNF-α (90). In the present study, pretreatment of IPEC-J2 with 5, 10, and 20 μg/mL DFP-NP1 followed by modeling with LPS achieved a better preventive effect by lower polysaccharides concentration, reducing the content and expression levels of *TNF-*α, *IL-1β*, and *IL-6*.

#### 3.5.2 Therapeutic effect of *Dendrobium findlayanum* polysaccharides on LPS-induced inflammation in IPEC-J2 cells

To assess the potential therapeutic effect of *D. findlayanum* polysaccharides on LPS-induced inflammation model, an inflammation model was first created using LPS on IPEC-J2 cells and then treated by *D. findlayanum* polysaccharides, followed by assessing the oxidative stress levels and expression of relevant inflammatory factors.

**Figure 6:**
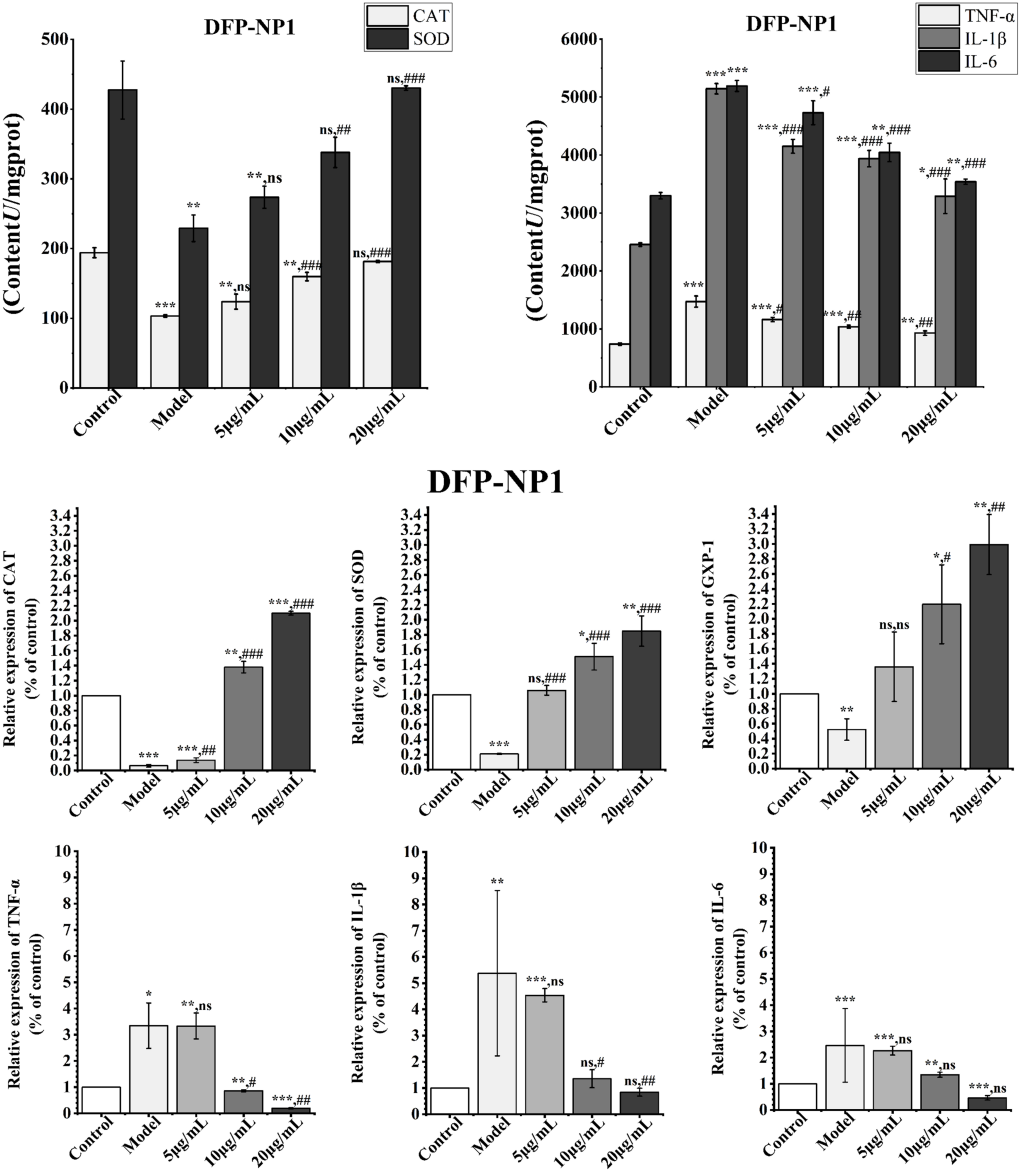
Effects of DFP-NP1 on CAT and SOD contents in IPEC-J2 inflammatory cells **(A)**; Effect of DFP-NP1 on TNF-α, IL-1β and IL-6 contents in IPEC-J2 inflammatory cells **(B)**; Effect of DFP-NP1 on *CAT*, *SOD*, *GXP-1*, *TNF-α*, *IL-1β*, and *IL-6* expression in IPEC-J2 inflammatory (C). (*n* = 5) (* vs control; # vs model; ns represents no significant difference).

Compared with the control group, LPS treatment significantly increased the intracellular oxidative stress level and TNF-α, IL-1β, and IL-6 protein content (*p* < 0.001), and *D. findlayanum* polysaccharides treatment significantly reduced the content of these three inflammatory factors, and the inhibitory effect was more pronounced with the increase of the concentration (Figure 6B); it is noteworthy that 20 μg/mL DFP-NP1 restored the SOD and CAT protein contents above the control level (*p* < 0.001, Figure 6A), suggesting its potential to repair oxidative damage. Similar phenomena have been reported in polysaccharide studies of other species of the genus *Dendrobium* (91), which may be related to acetylation modifications shared by *Dendrobium* polysaccharides, acetylation enhances polysaccharide bioactivity (e.g., antioxidant and anti-inflammatory effects) by increasing solubility and exposing reactive groups (e.g., hydroxyls), thereby improving interactions with cellular targets (92).

These findings were corroborated by q-PCR results, which showed that *CAT, SOD*, and *GXP-1* expression levels increased with rising polysaccharide concentrations (Figure 6C). This suggests that *D. findlayanum* polysaccharides possess potent antioxidant properties, capable of counteracting oxidative stress by upregulating the expression of *CAT*, *SOD*, and *GXP-1*. To further elucidate the expression levels of inflammatory factors in IPEC-J2 cells following DFP-NP1 treatment, q-PCR was employed to quantitatively detect *TNF-*α, *IL-1β*, and *IL-6*. *TNF-*α (*p* < 0.05), *IL-1β* (*p* < 0.01) and *IL-6* (*p* < 0.001) expression levels were significantly higher in the models than in the controls, indicating an inflammatory response in IPEC-J2 cells. DFP-NP1 at both 10 mg/mL DFP-NP1 and 20 mg/mL DFP-NP1 markedly decreased *TNF-*α and *IL-1β* gene expression, suggesting that *D. findlayanum* polysaccharides could help reduce inflammation-related damage by downregulating these pro-inflammatory markers.

DFP-NP1 restores the activity of antioxidant enzymes and inhibits the expression of pro-inflammatory factors in a dose-dependent manner, and its effects are closely related to molecular weight and structural modifications. However, its specific targets (e.g., whether it directly inhibits NF-κB or activates the Nrf2 pathway) still need to be further verified.

#### 3.5.3 *Dendrobium findlayanum* polysaccharides exert antioxidant effects by inhibiting release and activating the *Nrf2/HO-1/NQO1* pathway

The KEAP1/Nrf2-ARE pathway is a key antioxidant signaling mechanism that promotes the transcription of antioxidant genes, the KEAP1/Nrf2-ARE activates transcription of antioxidant genes by binding the ARE in the nucleus, mainly through the separation and translocation of Nrf2 from the repressor protein KEAP1 (93).

**Figure 7:**
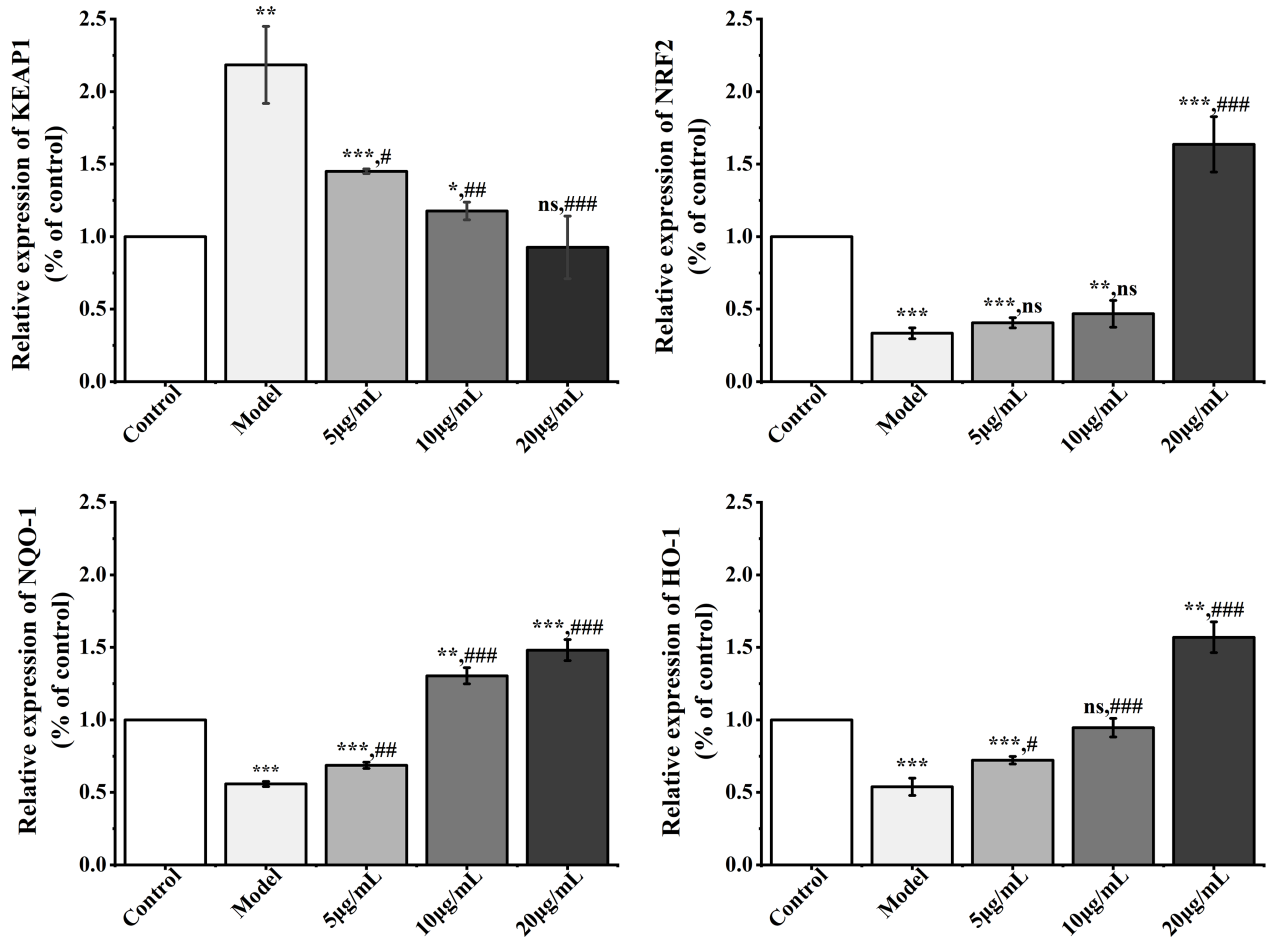
Effect of DFP-NP1 on genes related to oxidative stress pathway. (*n* = 5) (* vs control; # vs model; ns represents no significant difference).

In order to clarify the mechanism of modulation of antioxidant activity of polysaccharides from *D. findlayanum* on IPEC-J2 inflammatory cells, we examined the expression levels of genes in the KEAP1, Nrf2, NQO1, and HO-1 pathways, which are the upstream regulatory pathways of SOD1, GXP1, and CAT. The results are presented in Figure 7.

KEAP1 was significantly up-regulated (*p* < 0.01) and Nrf2 and its downstream effectors NQO1 and HO-1 were significantly down-regulated (*p* < 0.01) in the model compared to the control group, a phenomenon that suggests that LPS inhibits the activation of the antioxidant pathway by enhancing the ubiquitination degradation of Nrf2 by KEAP1, leading to the accumulation of intracellular oxidative stress (94). Compared to models, *D. findlayanum* polysaccharide significantly inhibited KEAP1 expression at 5 mg/mL DFP-NP1 (*p* < 0.05), and further inhibited it 10 mg/mL DFP-NP1 and 20 mg/mL DFP-NP1 (*p* < 0.01, *p* < 0.001); *Nrf2* expression was significantly upregulated at 20 mg/mL DFP-NP1 (*p* < 0.001), and *NQO1* was significantly upregulated at 10 mg/mL DFP-NP1 and 20 mg/mL DFP-NP1 (*p* < 0.01, *p* < 0.001); notably, HO-1 was more sensitive to DFP-NP1 and was already significantly activated at 5 μg/mL (*p* < 0.05), suggesting that HO-1 may be a preferentially regulated target in this pathway.

In summary, the model demonstrated upregulation of KEAP1 and downregulation of *Nrf2*, *NQO1*, and *HO-1* genes, inducing cellular inflammatory responses. DFP-NP1 reduced KEAP1 gene expression compared to the model while increasing *Nrf2*, *NQO1*, and *HO-1* gene expression. The activating effect of DFP-NP1 on the *Nrf2*/*HO-1*/*NQO1* pathway is related to its unique structural features, and the acetylation modification of DFP-NP1 (→4)-2-*O*-Ac-*β*-Man*p*-(1→) may be responsible for its better antioxidant activity, the modification of *Ganoderma applanatum* polysaccharides by acetylation modulates the *Nrf2*/*Keap1*-*TLR4*/*NFκB*-*Bax*/*Bcl-2* signaling pathway network, thereby attenuating oxidative stress and subsequent inflammatory responses (95).

#### 3.5.4 *Dendrobium findlayanum* polysaccharides exert anti-inflammatory effects through activation of the *TLR4/NF-κB* pathway

Excessive oxidative stress may lead to activation of *NF-κB* through *TLR4* signaling, resulting in a sustained and intense inflammatory response (96). Toll-like receptor 4 (*TLR4*) is a crucial receptor that recognizes polysaccharides, initiating an intracellular signaling cascade that produces pro-inflammatory factors by receptor cells (97). It has been shown that polysaccharides from *Fructus mori* and *Echinacea purpurea* activate *TLR4/NF-κB* signaling pathways, thereby mitigating LPS-induced inflammatory responses (98, 99).

**Figure 8:**
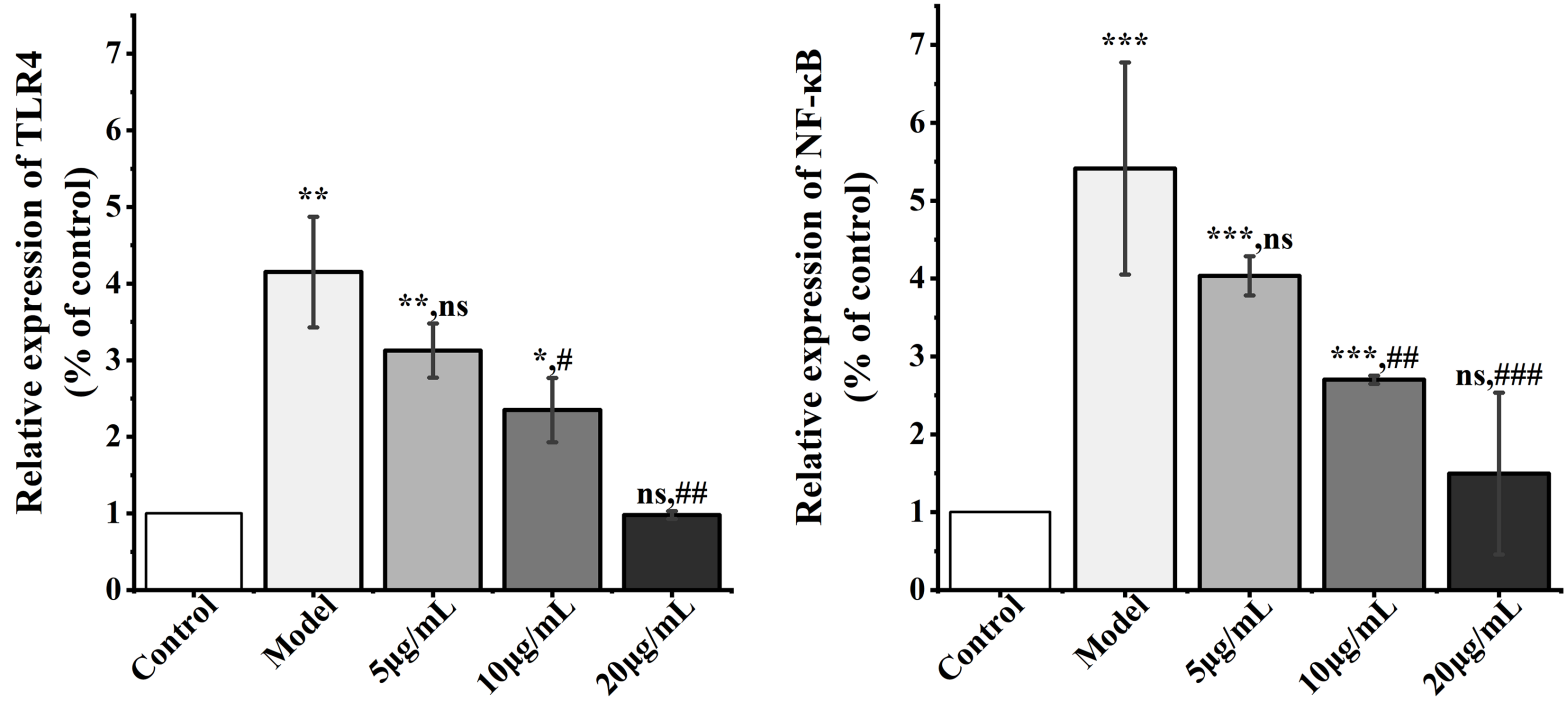
Effect of DFP-NP1 on anti-inflammatory pathway genes. (*n* = 5) (* vs control; # vs model; ns represents no significant difference).

To elucidate the mechanism by which *D. findlayanum* polysaccharides regulate the anti-inflammatory activity of IPEC-J2 inflammatory cells, we analyzed the expression levels of genes in the upstream regulatory pathways of *TLR4* and *NF-κB* signaling pathways (Figure 8).

LPS triggers the downstream *NF-κB* signaling pathway through activation of the *TLR4* receptor, which drives the expression of pro-inflammatory factors (*TNF-*α, *IL-1β*, *IL-6*), a phenomenon that has been demonstrated in both *Echinacea* polysaccharides (99) and *Polygonatum* polysaccharides (100). To elucidate the mechanism by which *D. findlayanum* polysaccharides modulate the anti-inflammatory activity of IPEC-J2 inflammatory cells as the *TLR4/NF-κB* pathway, we examined the expression levels of *TLR4* and *NF-κB* genes (Figure 8).

*TLR4* expression was significantly upregulated (*p* < 0.01) and *NF-κB* expression was significantly increased (*p* < 0.001) in the model compared to the control. Compared to the model, 5 mg/mL DFP-NP1 treatment did not significantly alter *TNF-*α expression. On the contrary, 10 mg/mL DFP-NP1 treatment significantly decreased *TNF-*α expression (*p* < 0.05), and 10 and 20 mg/mL DFP-NP1 treatment further increased *TNF-*α expression (*p* < 0.01).

Collectively, these findings suggest that DFP-NP1 achieves anti-inflammatory effects at the transcriptional level by modulating the *TLR4/NF-κB* pathway. The observed dose-dependent response suggests that higher concentrations of *D. findlayanum* polysaccharides are more effective in attenuating the inflammatory response by down-regulating important inflammatory mediators, a mode of action that is highly consistent with those reported for other polysaccharides of the genus *Dendrobium* (101, 102). However, the use of LPS as an inflammation inducer alone may only reflect the regulatory role of the TLR4/NF-κB pathway, and it cannot be excluded whether polysaccharides are effective on other inflammatory pathways (e.g., the TNF-α/IL-1β-mediated JAK–STAT or MAPK pathways), and therefore subsequent in-depth studies are needed.

Polysaccharides can be used as a core ingredient in functional foods for the development of dietary supplements targeting intestinal health [irritable bowel syndrome, inflammatory bowel disease (103)], and DFP-NP1 holds significant potential as a candidate formulation for promoting intestinal health with high *in vivo* application prospects. Following oral administration, this substance may act locally within the gastrointestinal tract, directly strengthening the intestinal barrier and alleviating inflammation through a dual-targeting mechanism. Furthermore, its polysaccharide properties suggest prebiotic potential, potentially promoting beneficial metabolites like short-chain fatty acids through gut microbiota regulation. With its natural origin from *Dendrobium* orchids and established safety profile, DFP-NP1 emerges as an ideal candidate for developing functional foods and dietary supplements supporting intestinal health and managing gut inflammation.

## 4 Conclusion

In this study, DFP-NP, a neutral polysaccharide with a molecular weight of 12.3 kDa, was isolated and purified for the first time from *D. findlayanum*. The polysaccharide was mainly composed of glucose (84.78%) and mannose (11.61%), and its structural features included: the presence of the →4)-α-Glcp-(1 → backbone and the Glcp-(1 → straight chain; the presence of special glycosidic bonds such as the acetylation-modified →4)-2-*O*-Ac-β-Manp-(1 → and →4)-3-*O*-Ac-β-Manp-(1 → and other special glycosidic bonds; the higher-order conformation of the polysaccharide, which combines both α/β conformation and branching structure, was elucidated by NMR for the first time. Notably, DFP-NP exhibited dual biological activities of therapy and prevention in the LPS-induced inflammation model of IPEC-J2 cells: DFP-NP alleviated oxidative stress via ROS scavenging and concurrently suppressed pro-inflammatory factors (e.g., TNF-αand IL-6; *p* < 0.01). These results suggest DFP-NP is a compelling candidate for dietary strategies designed to support intestinal well-being and combat inflammation through nutritional means.

Although the present study elucidated the structural characteristics and *in vitro* activity of DFP-NP, its specific target and molecular mechanism of action have not been clarified. Follow-up studies are needed to analyse the polysaccharide-receptor interactions and establish a model of the conformational relationship between structural modification and activity regulation, laying the foundation for developing *D. findlayanum* polysaccharides as a targeted functional food ingredient for gut health maintenance.

## Data Availability

The original contributions presented in the study are included in the article/Supplementary material, further inquiries can be directed to the corresponding author/s.
